# A Flexible Magnetic Field Sensor Based on AgNWs & MNs-PDMS

**DOI:** 10.1186/s11671-018-2826-5

**Published:** 2019-01-17

**Authors:** Qiang Zhang, Yi Du, Youyi Sun, Kai Zhuo, Jianlong Ji, Zhongyun Yuan, Wendong Zhang, Shengbo Sang

**Affiliations:** 1 MicroNano System Research Center, Key Laboratory of Advanced Transducers and Intelligent Control System of Ministry of Education and Shanxi Province & College of Information Engineering, Taiyuan University of Technology, Taiyuan, 030024 People’s Republic of China; 2 Technology of Polymeric Composites of Shanxi Province, North University of China, Taiyuan, 030051 People’s Republic of China

**Keywords:** Nanomaterials, Flexible sensor, Magnetic field detection

## Abstract

This paper presents a new flexible magnetic field sensor based on Ag nanowires and magnetic nanoparticles doped in polydimethylsiloxane (AgNWs & MNs-PDMS) with sandwich structure. The MNs act as the sensitive unit for magnetic field sensing in this work. Besides, the conductive networks are made by AgNWs during deformation. Magnetostriction leads to the resistance change of the AgNWs & MNs-PDMS sensors. Furthermore, the MNs increase the conductive paths for electrons, leading to lower initial resistance and higher sensitivity of the resulting sensor during deformation. A point worth emphasizing is that the interaction of the AgNWs and MNs plays irreplaceable role in magnetic field sensing, so the resistance change during stretching and shrinking was investigated. The flexible magnetic field sensor based on the mass ratio of MNs and AgNWs is 1:5 showed the highest sensitivity of 24.14 Ω/T in magnetic field sensing experiment. Finally, the magnetostrictive and piezoresistive sensing model were established to explore the mechanism of the sensor.

## Background

Flexible electronic devices have recently attracted tremendous attention due to their facile interaction long-term monitoring capabilities [1–5]. They become one of the most prospective electrical sensors due to the advantages such as light weight, portable, excellent electrical properties, and high integration [6–11]. Indubitably, nanomaterials play irreplaceable role in flexible sensors due to their outstanding properties, for instance small sizes, surface effect, and quantum tunneling effect [12–14]. Based on resonant tunneling effect of nanomaterials, many researches focus on piezoresistive strain sensors whose resistances change with deformation [15–17]. One of the key applications of the soft strain sensors is flexible electronic skin, so multi-fictionalizations are the development trend of the sensors. Some reports declared adding temperature [18, 19] and humidity [20, 21] sensing modules in the strain sensing arrays.

Besides strain, temperature, and humidity sensing abilities, the electronic skin sensing arrays are badly in need of some new functions. In another word, more functions make the electronic skin more intelligent. Among the new functions, magnetic field sensing is a novel application. It has to mention that only the soft magnetic field sensor can be used as a module for electronic skin in the future. Owning to soft magnetic field sensors can be used in more complex areas based on its flexibility and elasticity, some researchers are working on this field [22–26]. Chlaihawi et al. prepared ME flexible thin film sensor for H_ac_ sensing applications [27]. Jogschies et al. investigated thin NiFe 81/19 polyimide layers for magnetic field sensing [28]. Tekgül et al. applied the CoFe/Cu magnetic multilayers on GMR sensors [29]. Melzer et al. reported flexible magnetic field sensors relying on the Hall effect [30]. A number of flexible optical magnetic field sensor have been studied as well [31–34]. Comparing with traditional magnetic field detectors, flexible magnetic field sensors are more convenient to apply and they are smaller and more suitable for detection in complex environments. However, the studies about soft magnetic field sensor facing muti-functional electronic skin have been rarely reported as far as we know.

Due to the excellent electronic and magnetic properties of the Ag NWs [35–37] and MNs (Ni-Fe) [38, 39] respectively, this paper proposes the design and measurement of flexible AgNWs & MNs-PDMS magnetic field sensors with sandwich structure based on magnetostrictive and piezoresistive effects. MNs were introduced as magnetic field-sensitive units in AgNWs-based piezoresistive strain sensor. The different magnetostrictive deformation of the AgNWs & MNs-PDMS-based sensor causes the different resistance variations. After characterization of the nanomaterials, three different mass ratios of MNs and AgNWs (AgNWs & MNs; 1:1, 1:2, 1:5) were used to prepare flexible magnetic field sensors. Before the magnetic field sensing properties of the sensors were investigated, the relationships between resistance changes and stretching or retraction were studied to conclude the interaction of MNs and AgNWs. Based on the characterization results, the magnetic field sensor obtained in this work can be applied on muti-functional electronic in the future.

## Methods

### Preparation of Flexible Sensors

**Fig. 1:**
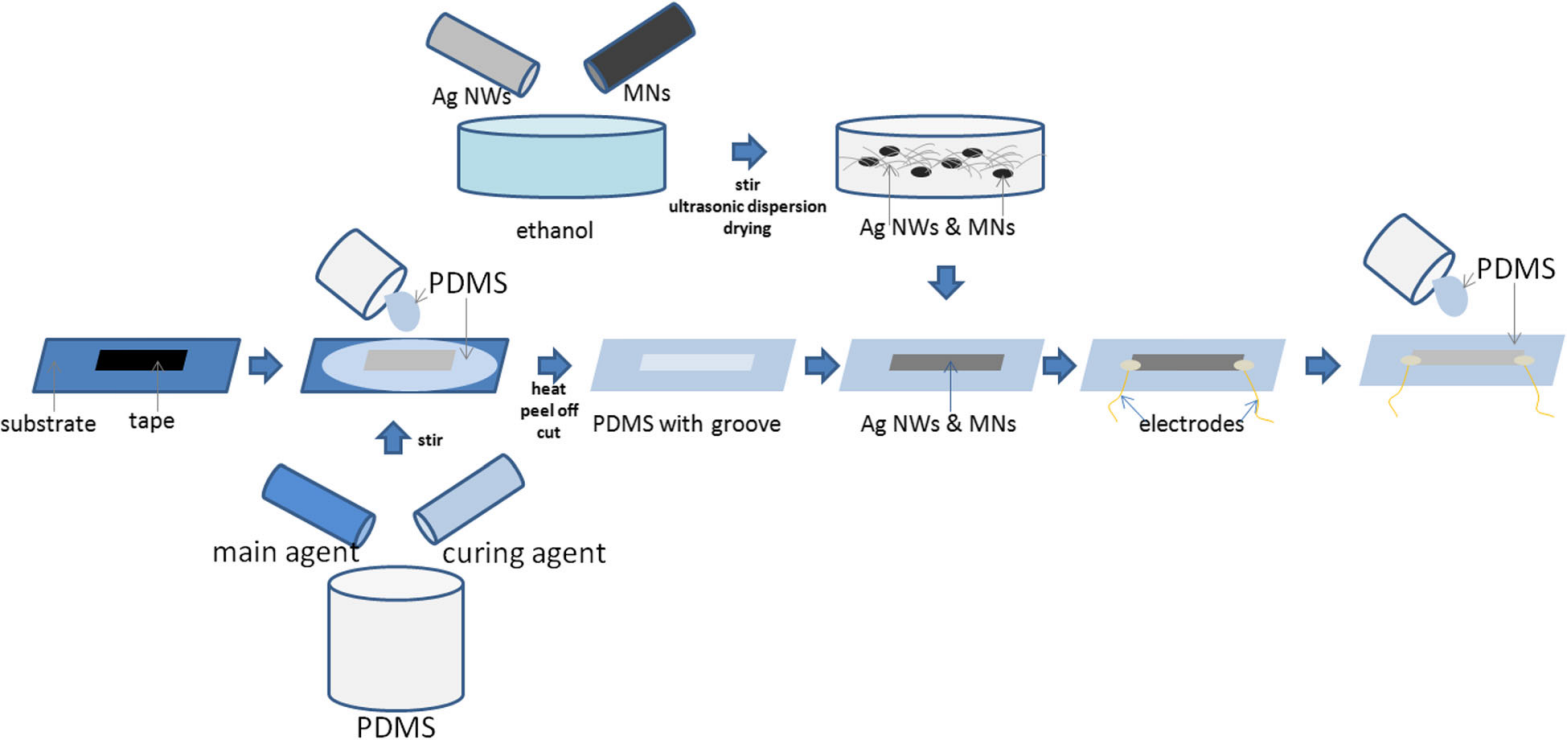
Schematic of the structural design and fabrication process flowchart of the sensor

MNs were synthesized by latex compounding method [24, 25]. The diameter and length of the AgNWs (which were purchased from the Changsha Weixi New Material Technology Corporation, China, in length) are 50 nm and 20 μm, respectively. Different ratios of MNs and AgNWs were chosen to investigate the proper amount of the nanomaterials. Thus, MNs and AgNWs in mass ratio of 0:1, 1:5, 1:2, and 1:1 were ultrasonic dispersed in absolute ethanol. Figure 1 shows the schematic of the fabrication process of the sensor. The PDMS elastomer and cross-linker in mass ratio of 10: 1 was dropped on the substrate with a rectangular tape pasted. After heated at 70 °C for 2 h, the PDMS with groove was peeled off and cut into required shape, and the groove size is 30 mm × 5 mm. Four samples of AgNWs & MNs in different ratios were filled in the notches of the PDMS films respectively. Two soft copper electrodes were installed on both sides, and then the PDMS was dropped on the top to fix the electrodes and nanomaterials. After heated at 70 °C for 2 h, the sensors were obtained.

### Characterization

AgNWs & MNs with different mixing ratios were characterized via scanning electron microscope (SEM, S4700 SEM Hitachi Corporation, Tokyo, Japan). The components of AgNWs & MNs in different mass ratios were characterized by XRD measurements (Buker D8 Advance) using Cu K radiation of wavelength 1.5406 Å.

The current-voltage curves were measured by the Keithley 2400 Source Meter at room temperature (the room temperature was 25 °C). Stretching experiments were carried out on the stretching platform (Zolix TSM25-1A and Zolix TSMV60-1 s, Zolix Corporation, Beijing, China), and the resistance of the sensors was measured by Keithley 2400 Source Meterat. Magnetic field sensing experiments were taken when the flexible sensor was fixed in different magnetic field. The magnetic field intensity started from 0 T and increases by 0.1 T.

## Results and Discussion

**Fig. 2:**
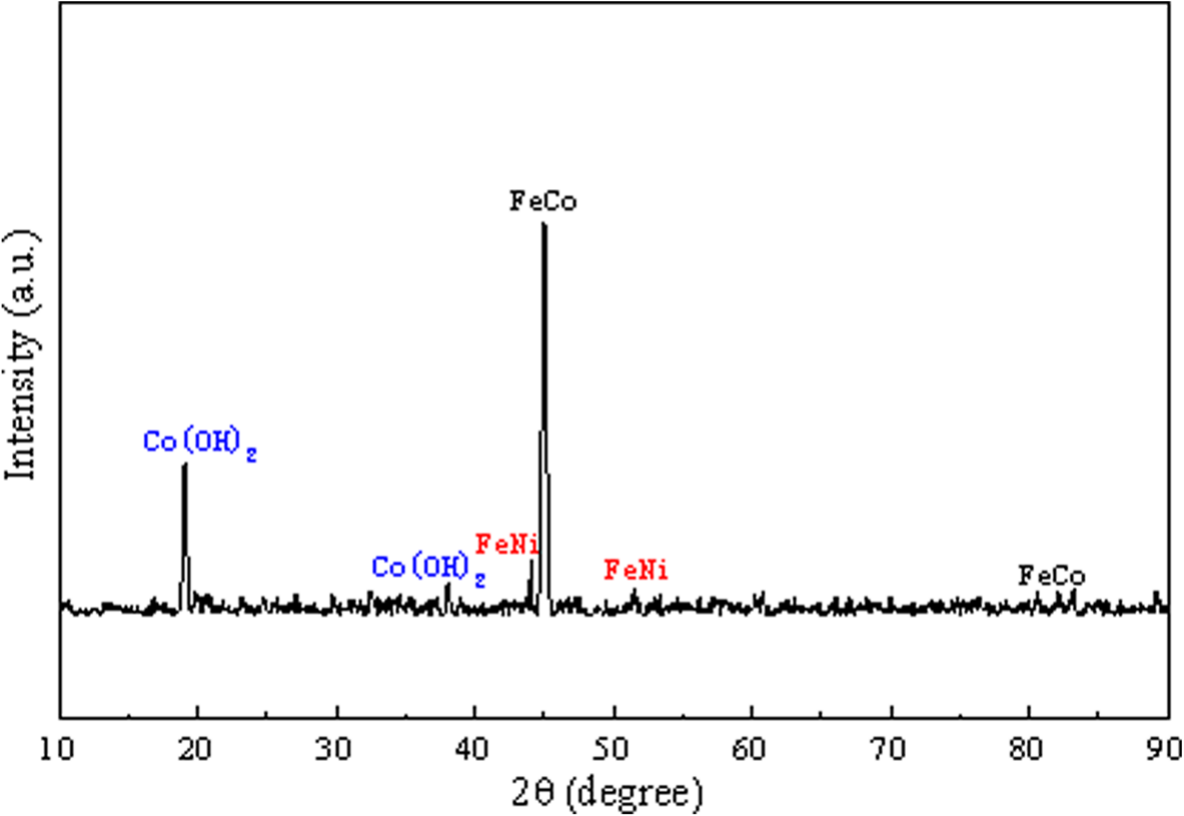
The XRD spectrum of MNs

The XRD spectrum of MNs was shown in Fig. 2. The characteristic peaks suggest that the MNs are composed with FeCo, FeNi, and Co(OH)_2_. The result demonstrates that all these compositions are magnetic materials.

**Fig. 3:**
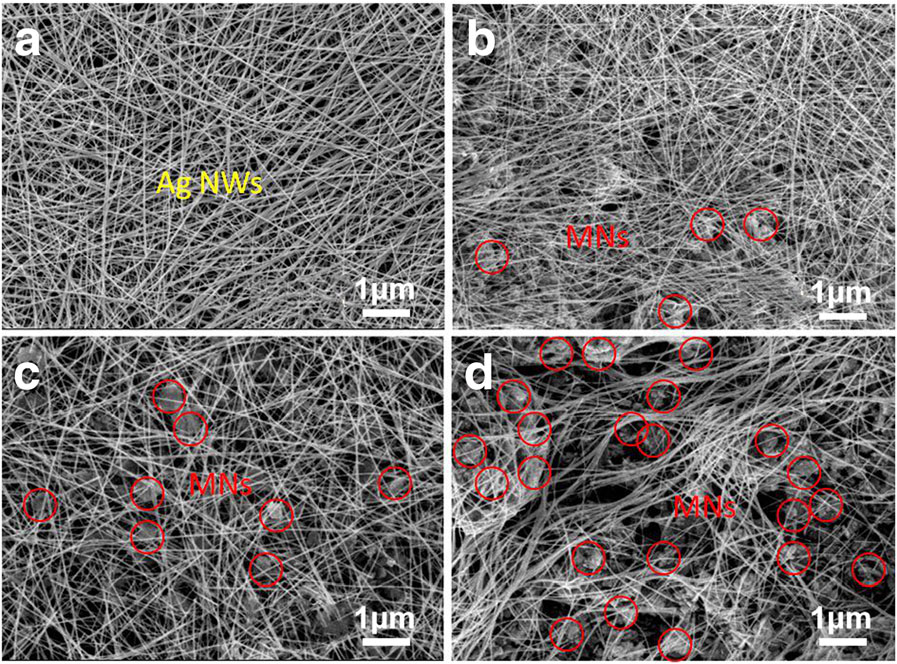
**a** AgNWs & MNs in mass ratio of 1:0, **b** 5:1, **c** 2:1, and **d** 1:1

The SEM images of AgNWs & MNs are displayed in Fig. 3. The pure Ag NWs with 20 μm in length and 50 nm in diameter form a linear network which can be observed in Fig. 3a. The morphologies of AgNWs & MNs in mass ratio of 5:1, 2:1, and 1:1 are exhibited in Fig. 3b–d. Small amounts of MNs among Ag NWs can be observed in Fig. 3b. The networks in Fig. 3c are sparser compared with Fig. 3a, b obviously. Moreover, the bending of the AgNWs and more MNs can be seen in Fig. 3d. The conductive networks which are built by AgNWs and the amount of MNs increase apparently in Fig. 3a–d. Uniform mixing Ag NWs and MNs, which are shown in Fig. 3a–d, play a connecting role for increasing sensors’ sensitivity when stretching or shrinking. The roles AgNWs and MNs played can be accounted by the results in Fig. 3.

**Fig. 4:**
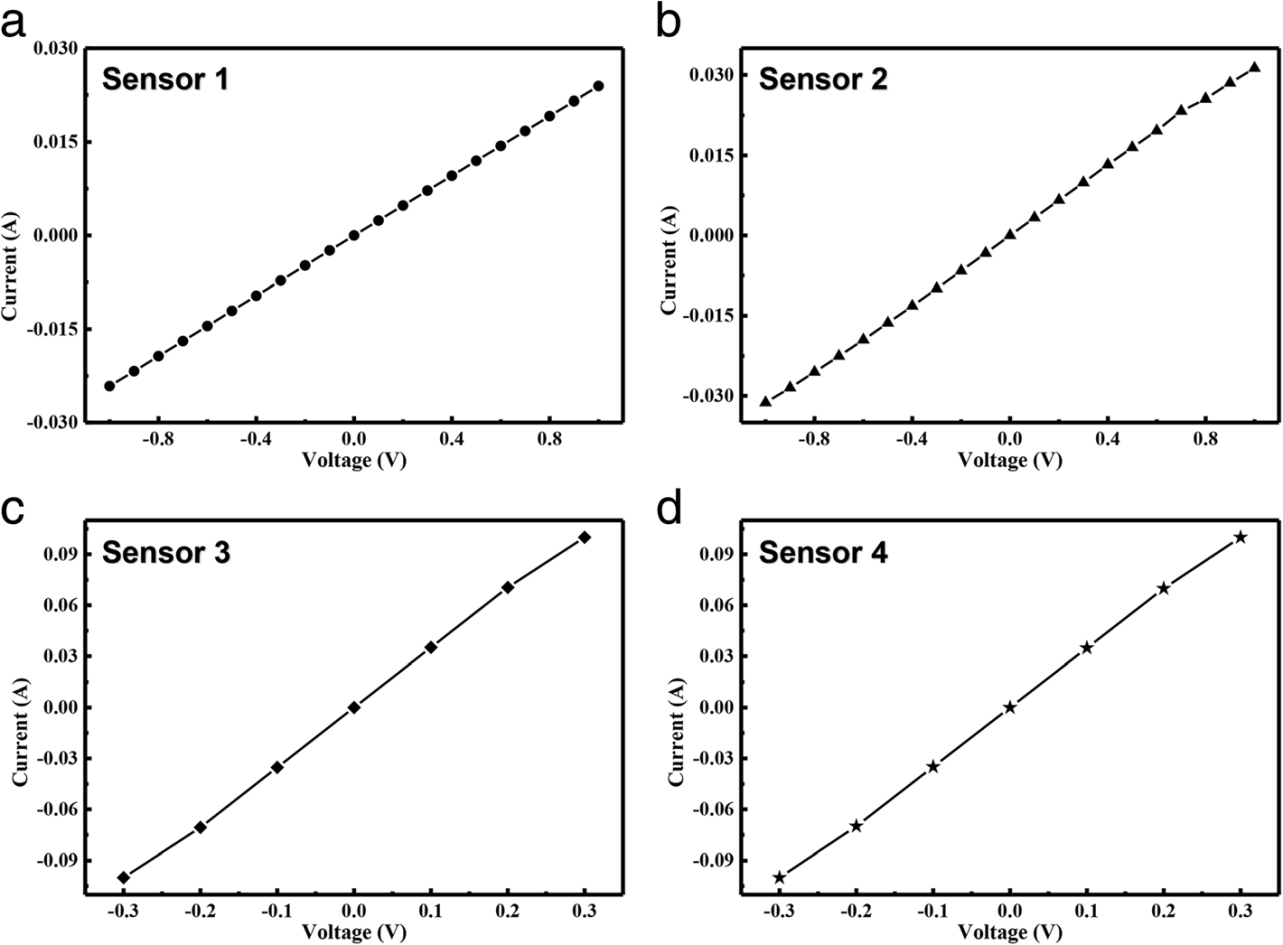
I-V curves of the sensors based on AgNWs & MNs in mass ratio of **a** 1:0, **b** 5:1, **c** 2:1, and **d** 1:1

The I-V curves of the sensors based on AgNWs & MNs in mass ratio of 1:0, 5:1, 2:1, and 1:1 are shown in Fig. 4. The four curves are all smooth straight lines, which represent the four sensors show significant ohmic characteristics. It declares that these sensors are conductive and stabile without deformation.

It can be calculated from Fig. 4a that the resistance of the sensor is 41.58  Ω when the sensitive unit is pure AgNWs. The resistances of the sensors based on AgNWs & MNs in mass ratio of 1:0, 5:1, 2:1, and 1:1 are 30.2 Ω, 5.04 Ω, and 2.87 Ω as shown in Fig. 4b–d. It shows a decreasing resistance trend when MNs were introduced into sensitive cells. Comparing the resistances of the four sensors, it can be concluded that the resistances of flexible magnetic field sensors decrease with the increasing proportion of MNs, and the minimal resistance occurs at the sensor with AgNWs & MNs in mass ratio of 1:1. It can also prove that the mixing of AgNWs & MNs in a certain proportion helps to reduce the resistance, because the conductive components of the MNs led more conductive paths in the networks.

**Fig. 5:**
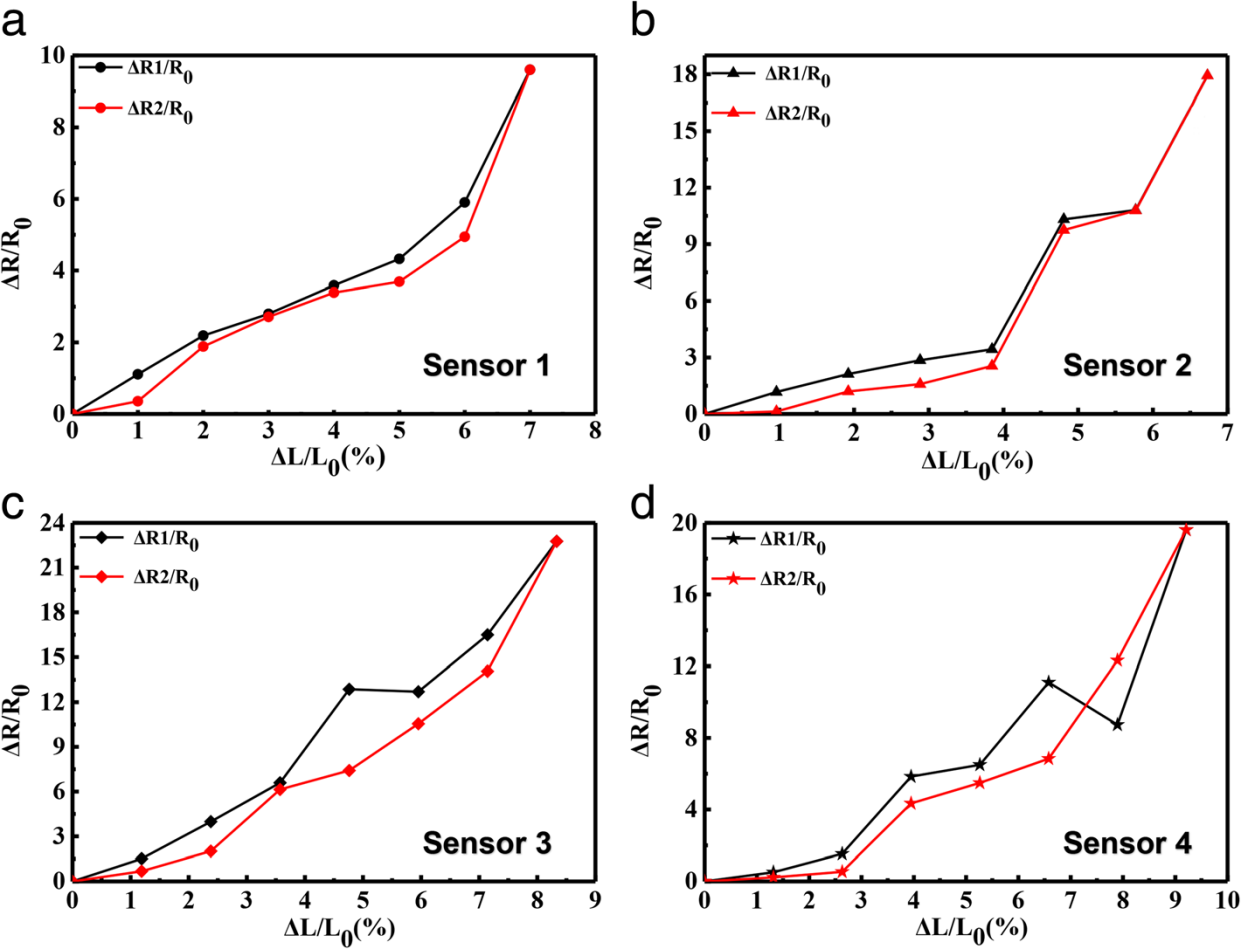
The relative resistance changes of the sensors based on AgNWs & MNs in mass ratio of **a** 1:0, **b** 5:1, **c** 2:1, and **d** 1:1 with deformation

The relationships between resistance changes, and stretching or retraction were studied to conclude the interaction between MNs and AgNWs during deformation. The relative resistance changes of the AgNWs & MNs-based sensors with extension under room temperature are shown in Fig. 5a–d. The resistance change during the stretching process is represented by black curves, and the change of resistance during the release process is plotted by red curves. Δ*R* and *R*_0_ represent the relative resistance change under the deformation and the initial resistance of the sensor, and *L*_0_ and Δ*L* represent the initial length and the relative elongation of the axial specimen of the sensor. The gauge factor of the sensors could be calculated through the equation of gauge factor (GF) = Δ*R*/*R*_0_: Δ*L*/*L*_0_. Figure 5a shows that the AgNWs-based sensor is conductive in the stretching and recovery process when the tensile length is within 7.12% of the original length, and its GF is 129.6. The resistance increases during stretching. This can be attributed to the increase in the spacing between AgNWs in the sensor during deformation, tunneling channels, and conductive path reduces in this way. The reverse process caused the decreasing of the resistance during retraction. When the MNs were introduced into the sensitive unit, the strain sensing characteristics of the flexible device also change. The resistance of the sensor based on AgNWs & MNs in mass ratio of 5:1 changes almost linearly when the stretching range is within 4.4% of the original length in Fig. 5b. When the tensile length is more than 3.9% of the original length, the higher increases of resistance occurred. The GF of the sensor increases to 257, which means the sensitivity of the sensor increased comparing with the sensor based on pure AgNWs. However, the strain range is not improved by MNs participation in mass ratio of 5:1, which can be observed in Fig. 5a, b. Figure 5c demonstrates that the resistance of sensor based on AgNWs & MNs in mass ratio of 2:1 changes linearly when the stretching range is within 8.7% of the original length, and the GF of the sensor is 264.4, which is higher than that of the sensors based on AgNWs & MNs in mass ratio of 1:0 and 5:1. In Fig. 5d, the resistance of the sensor based on AgNWs & MNs in mass ratio of 1:1 changes linearly when the stretching range is within 9% of the original length. When the tensile length is more than 9% of the original length, the resistance changes substantially, and the GF is 222.2. In summary, the flexible magnetic field sensor based on AgNWs & MNs in mass ratio of 2:1 shows largest GF of 264.4, and it has relatively large stretchable range. Moreover, this sensor responds more sensitively as stress increases, the resistance change has a better linear relationship as well. Based on the main ingredient of the MNs is FeCo, which is conductive alloy. Comparing these four kinds of sensors, the more MNs’ participation makes more conductive paths in the sensitive units during stretching. However, higher ratio of MNs in Ag NWs &MNs in same quality means less amount Ag NWs existence, which is harmful for the stability of conductive network during deformation. That is the reason of the relative resistance plunge at 9% displacement. Consequently, the AgNWs & MNs in mass ratio of 1:1 is the highest MNs amount we designed in this work, and the sensor based on the AgNWs & MNs in mass ratio less than 1:1 is non-conductive as soon as stretching. The results of the Fig. 5 demonstrate that the synergistic effects of the AgNWs and MNs in certain ratios increase sensitivity and strain range.

**Fig. 6:**
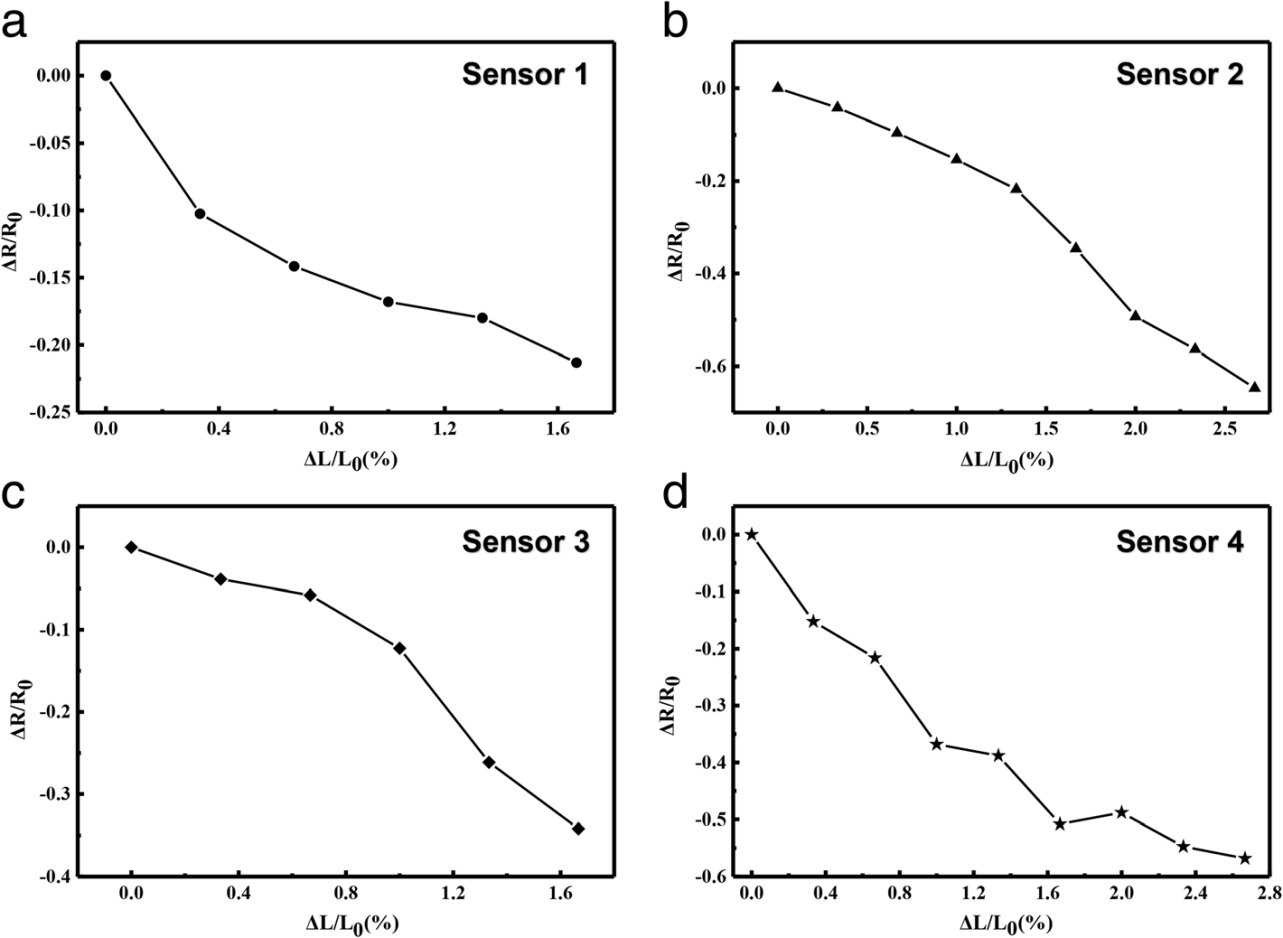
The relative resistance changes of the sensors based on AgNWs & MNs in mass ratio of **a** 1:0, **b** 5:1, **c** 2:1, and **d** 1:1 with shrinkage

The MNs may move closer under magnetic field, so magnetostriction may lead to the shrink of the sensors. In order to characterize the interaction of AgNWs and MNs in the sensors during shrinking, we measured the resistance change during shrinking, and the experimental results are shown in Fig. 6. Figure 6a shows that the AgNWs-based sensor is conductive in the process of shrinking and recovery when the contraction length is within 1.6% of the original length, and its highest GF is 13.75; AgNWs embedded in PDMS contact together during shrinking process, which leads to the increase of conduction paths. Therefore, the resistance decreases as the contractile force increases. The decrease in the spacing between AgNWs in the sensor, more and more nanowires are overlapping, resulting in decreasing of the sensor’s resistance. When we introduced the MNs into AgNWs, Fig. 6b illustrates that the shrink characteristics of the flexible device based on the AgNWs & MNs in mass ratio of 5:1. The resistance of the sensor changes with the shrinking range is 2.5% of the original length, and the highest GF is 24. Substantially, the same change in resistance also applies to sensors based on the AgNWs & MNs in mass ratio of 2:1 and 1:1, which is shown in Fig. 6c, d. Increasing the mass ratio of MNs in sensitive unit, the resistance of sensor based on the AgNWs & MNs in mass ratio of 2:1 changes when the shrinking range is within 1.6% of the original length, and its GF is 21.875. At the same time, the resistance of sensors based on the AgNWs & MNs in mass ratio of 1:1 also decreased when the shrinking range is within 2.8% of the original length, and its GF is 20.35. It can be concluded that the resistance change of the sensor based on the AgNWs & MNs in mass ratio of 5:1 with shrink is larger than that of the other three sensors, and the sensitivity is largest. Contrary to the stretching process, the resistance of all sensors decreases as the length of the contraction increases. When AgNWs & MNs in mass ratio is 5:1, the sensor has the highest sensitivity coefficient during the contraction process, whose highest GF is 24. Comparing Fig. 6a–d, less amount of MNs connect the conductive paths easier because there are more space for the materials moving as shrinking, which is contrary to the results of Fig. 5. Accordingly, the GF of sensor based on the AgNWs & MNs in mass ratio of 5:1 is highest when shrinking. The results of the Fig. 6 demonstrate that the synergistic effects occurs when AgNWs and MNs at larger ratio.

**Fig. 7:**
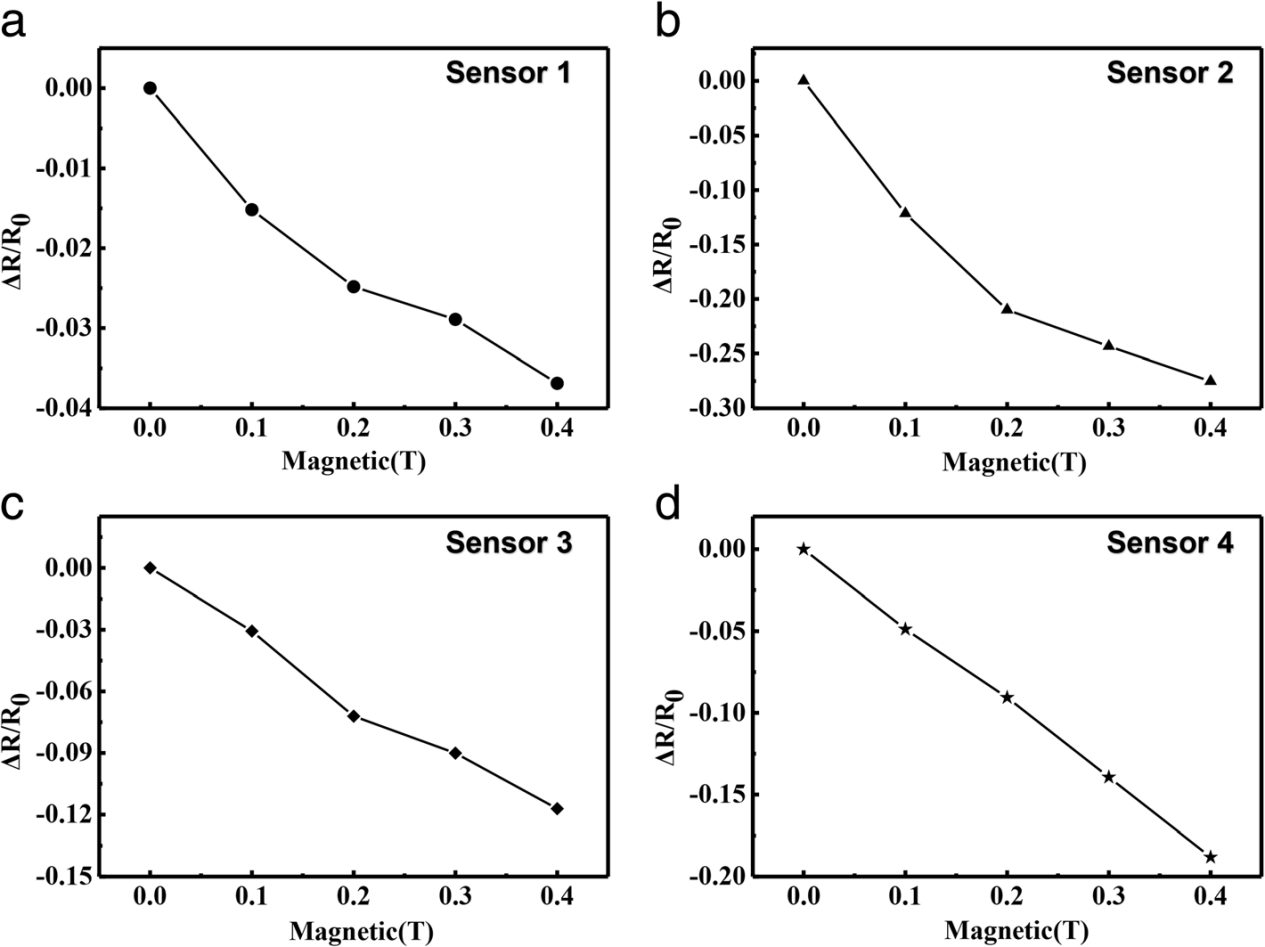
The resistance changes in different magnetic fields

**Fig. 8:**
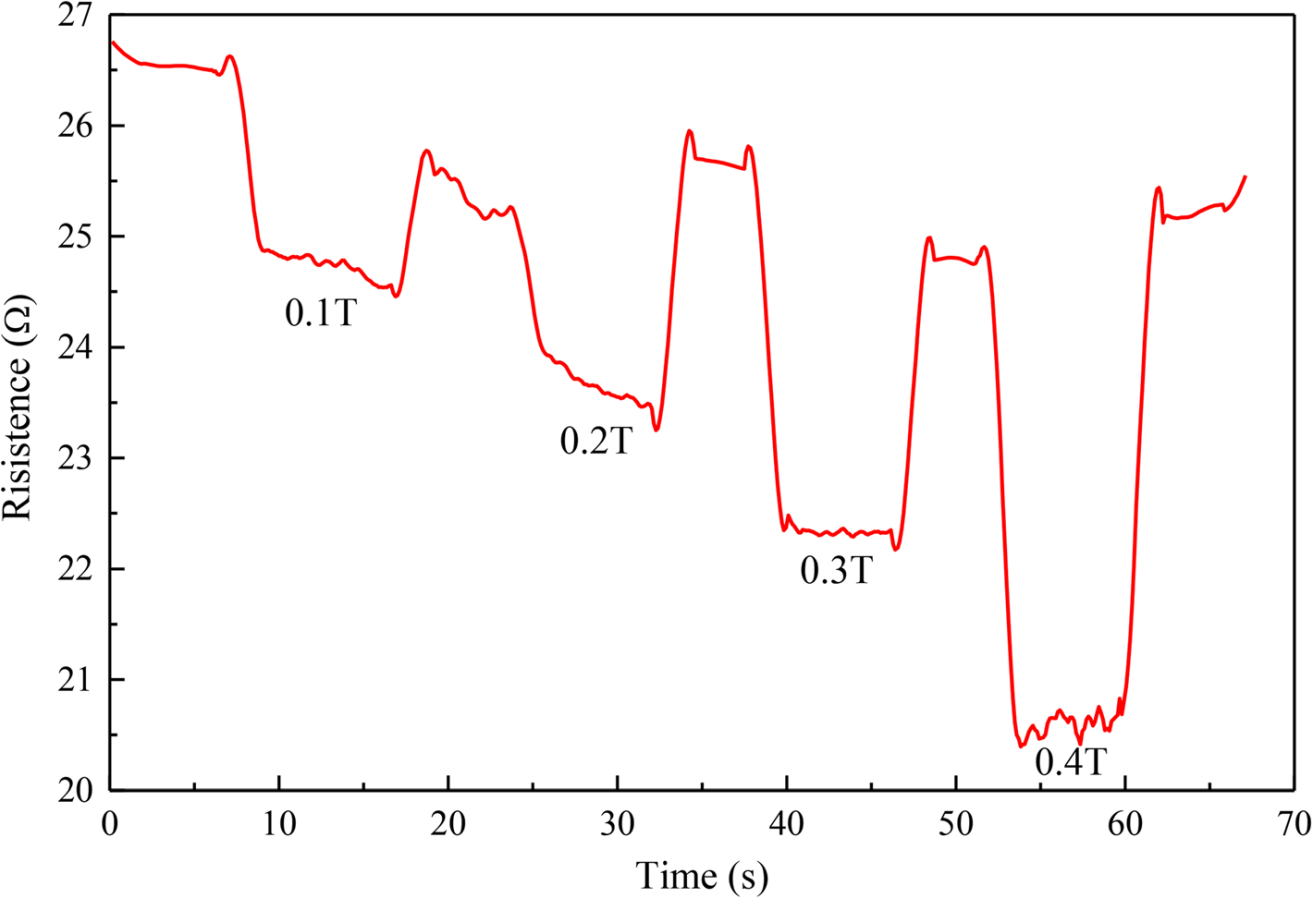
The relationship between resistance and different magnetic fields

In different magnetic fields, different flexible magnetic sensor resistance changes are shown in Fig. 7. The resistance of the AgNWs based sensor is 41.58 Ω. As shown in Fig. 7a, we put the sensor based on pure AgNWs in a gradually increasing magnetic field, and the resistance of the sensor changes as it vibrates accordingly. Due to the magnetostrictive effect of the metal materials, the resistance of the sensor is slightly changed. The maximum resistance change rate is 0.037 when the magnetic field strength is 0.4 T. The resistance of sensor based on the AgNWs & MNs in mass ratio of 5:1 also decreases with the magnetic field strength increasing as shown in Fig. 7b. Compared with the sensor without MNs, the resistance change of the sensor based on the AgNWs & MNs in mass ratio of 5:1 with magnetic field change is more obvious. When the magnetic field strength is 0.4 T, the maximum rate of resistance change is 0.28. In Fig. 7c, d, the same application to the sensors based on the AgNWs & MNs in mass ratio of 2:1 and 1:1, and the resistance changes are 0.14 and 0.19 as the magnetic field increases respectively. The sensitivity of the sensor based on the AgNWs & MNs in mass ratio of 5:1 is the highest, and the continuous resistance variation with magnetic field was shown in Fig. 8. The comparison of the parameters of the strain sensors based on different ratios of MNs and AgNWs is presented in Table 1.

**Table 1:** Parameters of the strain sensors based on different ratios of MNs and AgNWs

Mass ratio of MNs: AgNWs value	Resistance (Ω)	Stretching range (%)	GF	Shrinking range (%)	GF	∆R/R_0_ (magnetic field intensity = 0.4 T)
*0:1*	41.58	0–7.12	129.6	0–1.7	13.75	0.037
*1:5*	30.2	0–6.8	257	0–2.6	*24*	*0.28*
*1:2*	5.04	0–8.7	*264.4*	0–1.7	21.875	0.14
*1:1*	2.87	*0–9*	222.2	*0–2.7*	20.35	0.19

It can be calculated that the sensitivity of the magnetic field sensor is 24.14 Ω/T. In conclusion, when the mass ratio of MNs and AgNWs is 1:5, the sensor’s response of the changing magnetic field is most sensitive with a sensitivity of 24.14 Ω/T. The flexible magnetic field sensor obtained in this work can be further applied on detection of the intensity of magnetic field. The test results of this application are corresponding to the shrink process of the sensor when comparing the results in Figs. 7 and 8. This means that the nanomaterials in the sensors move together when they were put in magnetic field. The mechanism analysis declares in detail as following.

**Fig. 9:**
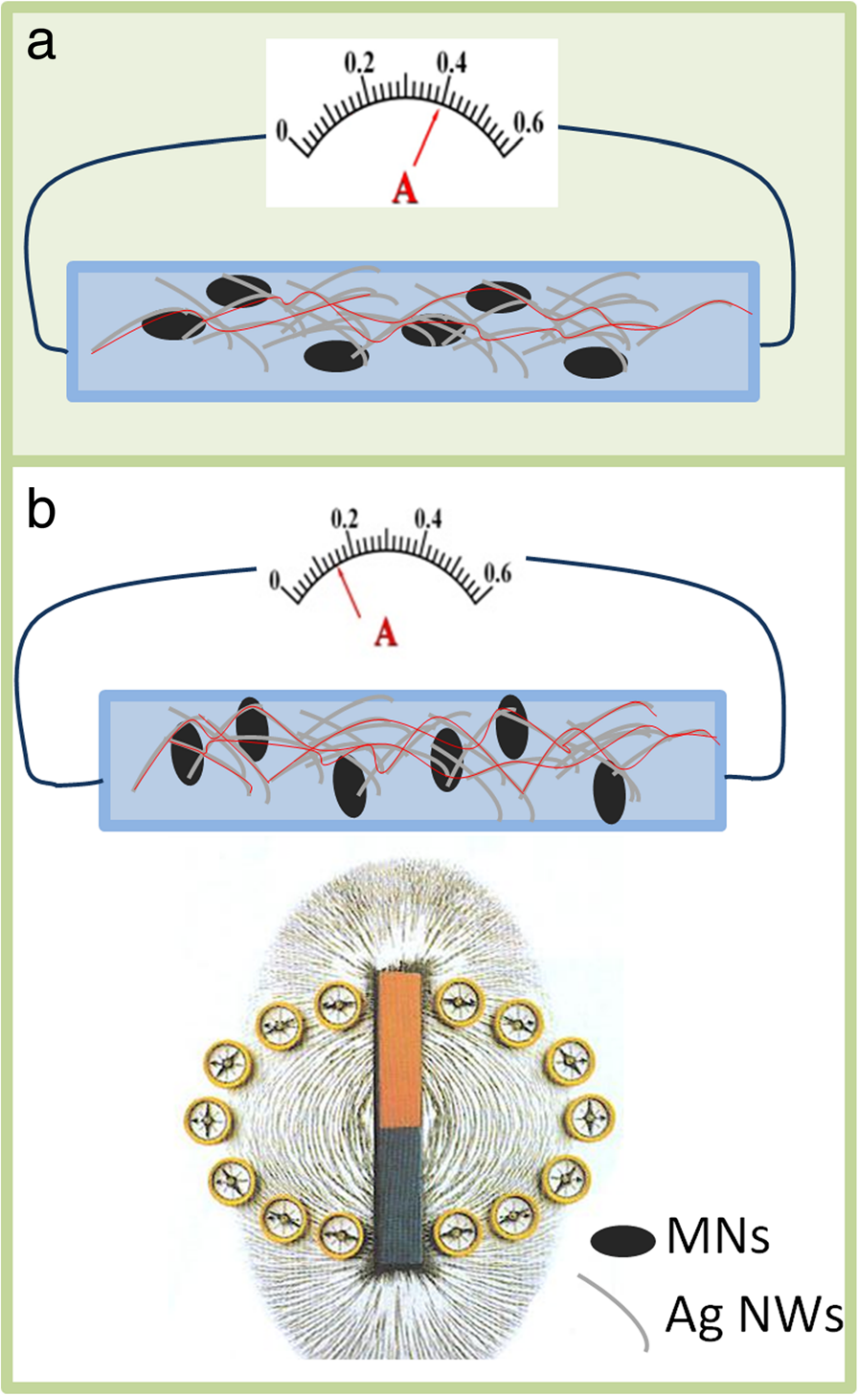
Schematic sensing model of AgNWs & MNs-PDMS-based soft magnetic field sensor

To understand the resistance variations of the sensors during different magnetic field intensity, we propose a simple model to describe the working principle of the sensor as shown in Fig. 9. Numerous AgNWs and MNs in PDMS form a conductive network. The conductive paths formed by AgNWs and MNs without magnetic field is shown as the red lines in Fig. 9a. The MNs tend to be uniformly arranged under magnetic field, which is shown in Fig. 9b. However, there is tiny space for the position change of the MNs, so only the directions of MNs change with magnetic field lines. The higher magnetic field intensity stands for larger force of the MNs that can overcome the network constraints of the AgNWs. The direction of the movement of the MNs makes the Ag NWs gather together, which is the reason for the increase in conductive paths’ number. More conductive paths mean more electrons transfer, which leads to lower resistance, the resistance decreases with the increase of magnetic field intensity in this way.

## Conclusions

The device designed in this paper conforms with the development trend of flexible electronics. A flexible magnetic field sensor based on AgNWs & MNs-PDMS with sandwich structure was studied in this work. Based on SEM and XRD characterizations, the components and morphologies of the different ratios of nanomaterials were determined. Then, the current-voltage curves and resistance changes of the sensors based on AgNWs & MNs in mass ratio of 1:0, 5:1, 2:1, and 1:1 with stretch and shrink were measured respectively. The interaction between the AgNWs and MNs during deformation was concluded through the characterization results. Then, sensors based on different mass ratio of MNs and AgNWs were investigated for magnetic field sensing properties. When the mass ratio of AgNWs and MNs is 5:1, the as-prepared sensor shows highest sensitivity of 24.14 Ω/T. The experimental results show that the sensor shrink with the magnetic field intensity increasing. Moreover, the magnetostrictive and piezoresistive sensing model were established to explore the mechanism of this sensor.

## Abbreviations

AgNWs: Ag Nanowires
GF: Gauge factor
MNs: Magnetic nanoparticles
PDMS: Polydimethylsiloxane
SEM: Scanning electron microscope
XRD: X-ray diffraction
